# Coronin-1C Protein and Caveolin Protein Provide Constitutive and Inducible Mechanisms of Rac1 Protein Trafficking*[Fn FN2]

**DOI:** 10.1074/jbc.M115.640367

**Published:** 2015-04-29

**Authors:** Rosalind C. Williamson, Christopher A. M. Cowell, Thomas Reville, James A. Roper, Thomas C. S. Rendall, Mark D. Bass

**Affiliations:** From the ‡ School of Biochemistry and; § Department of Engineering, University of Bristol, University Walk, Bristol BS8 1TD, United Kingdom and; the ¶ Centre for Membrane Interactions and Dynamics, Department of Biomedical Science, University of Sheffield, Western Bank, Sheffield S10 2TN, United Kingdom

**Keywords:** caveolin, endocytosis, fibroblast, fibronectin, migration, Rac (Rac GTPase), trafficking, coronin-1c, syndecan-4

## Abstract

Sustained directional fibroblast migration requires both polarized activation of the protrusive signal, Rac1, and redistribution of inactive Rac1 from the rear of the cell so that it can be redistributed or degraded. In this work, we determine how alternative endocytic mechanisms dictate the fate of Rac1 in response to the extracellular matrix environment. We discover that both coronin-1C and caveolin retrieve Rac1 from similar locations at the rear and sides of the cell. We find that coronin-1C-mediated extraction, which is responsible for Rac1 recycling, is a constitutive process that maintains Rac1 protein levels within the cell. In the absence of coronin-1C, the effect of caveolin-mediated endocytosis, which targets Rac1 for proteasomal degradation, becomes apparent. Unlike constitutive coronin-1C-mediated trafficking, caveolin-mediated Rac1 endocytosis is induced by engagement of the fibronectin receptor syndecan-4. Such an inducible endocytic/degradation mechanism would predict that, in the presence of fibronectin, caveolin defines regions of the cell that are resistant to Rac1 activation but, in the absence of fibronectin leaves more of the membrane susceptible to Rac1 activation and protrusion. Indeed, we demonstrate that fibronectin-stimulated activation of Rac1 is accelerated in the absence of caveolin and that, when caveolin is knocked down, polarization of active Rac1 is lost in FRET experiments and culminates in shunting migration in a fibrous fibronectin matrix. Although the concept of polarized Rac1 activity in response to chemoattractants has always been apparent, our understanding of the balance between recycling and degradation explains how polarity can be maintained when the chemotactic gradient has faded.

## Teaser

**Background:** Directional migration requires removal of Rac1 from the back of the cell.

**Results:** Coronin-1C and caveolin provide parallel pathways for constitutive recycling or fibronectin-inducible degradation of Rac1.

**Discussion:** Redundant trafficking pathways facilitate polarity while migrating, and responsiveness to chemotactic gradients while stationary.

**Significance:** Maintenance of polarized migration, when the chemotactic gradient has dissipated, is essential for wound healing over a period of days.

## Introduction

In adult animals, the migration of dermal fibroblasts is a tightly regulated process that is responsible for the organization of extracellular matrix and the contraction of wounds upon skin damage. Key regulation events include the initiation of migration but also the polarization of signals that cause fibroblasts to translocate over many cell lengths and cluster at the wound bed. As the regulator of membrane protrusion, Rac1 is a nexus in migration regulation, and inhibition or activation of Rac1 retards or accelerates skin healing, respectively (1–3). Historically, mechanisms of Rac1 regulation have focused on activation by guanine-nucleotide exchange factors and GTPase-activating proteins (4). More recently, the focus has shifted toward mechanisms for sequestration either in the cytoplasm by guanine-nucleotide dissociation inhibitors (GDIs)^2^ (5) or at membranes by RCC2 (6) and PACSIN2 (7) or localization of Rac1 by regulation of trafficking.

Localization of Rac1 plays an important role in the regulation of signaling. In the skin, engagement of the fibronectin receptors syndecan-4 and α_5_β_1_-integrin trigger the activation of Rac1 and are necessary for efficient wound healing (8). Syndecan-4, in particular, is very good at detecting dilute extracellular agonists but very poor at localizing the resultant Rac1 signal because it is a diffusely distributed transmembrane proteoglycan. Coupled with the facts that active Rac1 turns over rapidly and that fibronectin gradients are both shallow and transient, additional factors, such as protein localization, are necessary to sustain polarized signaling after the initial stimulation event. There are three major mechanisms for Rac1 redistribution. The original mechanism involves extraction of GTPases from the plasma membrane by RhoGDI for sequestration in the cytosol (9). The second mechanism involves caveolin-dependent endocytosis of Rac1 and is particularly interesting because caveolin distribution is polarized in fibroblasts and regulated by both integrins and syndecan-4 (10–13). Recently, we reported a third mechanism involving the coronin-1C (Coro1C)-dependent redistribution of Rac1 through the vesicular system (6). Like caveolin, Coro1C associated with particular membrane compartments, including the non-protrusive rear and sides of a migrating fibroblast, which were collectively termed the lateral membrane. The existence of such apparently similar mechanisms for the removal of Rac1 from the plasma membrane raises questions about how Coro1C and caveolin mechanisms are coordinated and whether they fulfill specific or redundant functions.

In this work, we discover that Coro1C and caveolin play distinct and complimentary roles in Rac1 trafficking. We discover that, although Coro1C redistributes Rac1 from the lateral membrane for reactivation regardless of syndecan-4-engagement, caveolin removes Rac1 from similar locations for proteasomal degradation. The degradative events are triggered by engagement of syndecan-4 by fibronectin, meaning that the fate of Rac1 depends on the matrix environment. We find that, between them, Coro1C and caveolin define regions of the membrane that are resistant to Rac1 signaling and, thereby, sustain processive, linear migration.

## Experimental Procedures

### 

#### 

##### Antibodies and Extracellular Matrix Proteins

Mouse monoclonal antibodies raised against Rac1; β_1_-integrin, EEA1, Rab4 (BD Transduction Laboratories), tubulin (catalog no. DM1A, Sigma), cortactin (Millipore), Coro1C (Abnova), HSP70 (Affinity BioReagents), and the α1 Na^+^ K^+^ ATPase transporter (Millipore) and polyclonal antibodies against dynamin-2 (Santa Cruz Biotechnology), caveolin (BD Transduction Laboratories), and flotillin-2 (Cell Signaling Technology) were used according to the instructions of the manufacturer. DyLight 680- and 800-conjugated IgGs were from Fisher, and Cy2- and Cy3-conjugated IgGs were from Stratech Scientific. Recombinant fibronectin polypeptides encompassing type III repeats 6–10 (50K) (14) and 12–15 (H/0) (15) were expressed as recombinant polypeptides as described previously (16).

##### Cell Culture

Immortalized wild-type and *Scd4*^−/−^ MEFs have been described previously (17) and were cultured at 33 °C in DME, 10% fetal bovine serum, 4.5 g/liter glucose, 2 mm l-glutamine, and 20 units/ml IFNγ (Sigma). *Cav1*^−/−^ MEFs, MEFs from wild-type littermates, and rescued MEFs (all gifts from M. Del Pozo (Centro Nacional de Investigaciones Cardiovasculares, Madrid) were cultured at 37 °C in DME, 10% fetal bovine serum, 4.5 g/liter glucose, and 2 mm l-glutamine. Telomerase-immortalized human fibroblasts transfected with GFP-RhoG were cultured at 37 °C in DME, 15% fetal bovine serum, 4.5 g/liter glucose, 25 mm HEPES, and 2 mm l-glutamine.

For RNAi, siRNA duplexes with ON TARGET^TM^ modification were transfected with Dharmafect2 reagent (Thermo Fisher Scientific). Sequences targeted the sense strand of mouse Coro1C (CCGUUGAAUUAAUUACGUA or GUAUAAACACUCACGAGAA), caveolin-1 (GCUAUUGGCAAGAUAUUCA and GUCCAUACCUUCUGCGAUC), human Coro1C (GCACAAGACUGGUCGAAUU), dynamin-2 (GAGAUCAGGUGGACACUCU), and caveolin-1 (GCAUCAACUUGCAGAAAGA) using siGLO as a control. GFP-Coro1C, GFP-Coro1A, GFP-Rac1-C*AAX*, and PAGFP-Rac1 have been described previously (6) and were transfected using FuGENE HD (Promega).

##### Cell Spreading for Biochemical Assays

9-cm tissue culture-treated plastic dishes (Corning BV) were coated overnight at 4 °C with 20 μg/ml 50K in Dulbecco's PBS containing calcium and magnesium (Sigma). Cells were treated with 25 μg/ml cycloheximide (Sigma) for 2 h to prevent *de novo* synthesis of extracellular matrix and other syndecan-4 ligands and then spread on the 50K-coated plates for 2 h in DME, 4.5 g/liter glucose, 25 mm HEPES, and 25 μg/ml cycloheximide. Spread cells were stimulated with 10 μg/ml H/0 for 0–60 min before preparing lysates.

##### Detergent Fractionation

Cells were lysed in 20 mm HEPES (pH 7.4), 10% (v/v) glycerol, 140 mm NaCl, 1% (v/v) Nonidet P-40, 4 mm EGTA, 4 mm EDTA, and complete protease inhibitor, and the detergent-insoluble pellet was separated from the soluble/vesicle fraction by centrifugation at 22000 × *g* for 5 min.

##### Mechanical Fractionation

Cells were harvested at 4 °C by scraping in Dulbecco's PBS containing calcium and magnesium, complete protease inhibitor (Roche), 5 mm Na_2_VO_4_, and 10 mm NaF. Membranes were fragmented by three 12-J pulses using a Vibra-Cell sonicator (Sonics) before removing nuclear debris with a 10-min, 1000 × *g* centrifugation step. Membranes were separated into a plasma membrane pellet and vesicle/soluble supernatant by 10-min centrifugation at 10,000 × *g*. For immunoprecipitation, ProteinG dynabeads (Invitrogen) and the appropriate antibody were incubated with unclarified lysate for 2 h before separation from unbound material by magnetic sorting.

##### Rac1 Degradation Assays

For degradation inhibition experiments, lysosomal degradation was blocked by overnight treatment with 100 nm bafilomycin A1 (R&D Systems), proteasomal degradation was blocked by 3-h treatment with 40 μm MG132 (Sigma), and protein synthesis was blocked by treatment with 25 μg/ml cycloheximide for 0, 2, or 20 h.

##### Rac1 Activation

Cells were lysed in 20 mm HEPES (pH 7.4), 10% (v/v) glycerol, 140 mm NaCl, 1% (v/v) Nonidet P-40, 0.5% (w/v) sodium deoxycholate, 4 mm EGTA, 4 mm EDTA, and complete protease inhibitor. GST-PAK-CRIB (p21-activated kinase Cdc42 and Rac interacting binding domain)-loaded agarose beads were incubated with the lysates for 1 h and then washed three times with lysis buffer.

##### Immunofluorescence

For experiments on two-dimensional fibronectin, 13-mm-diameter glass coverslips were coated with 10 μg/ml fibronectin (Sigma) in Dulbecco's PBS containing calcium and magnesium (Sigma). For experiments on three-dimensional CDM, coverslips were prepared as described previously (17). Cells were spread for 4 h in DME, 10% FBS, 4.5 g/liter glucose, and 25 mm HEPES; fixed with 4% (w/v) paraformaldehyde; permeabilized with 0.5% (w/v) TrX diluted in PBS; and blocked with 3% (w/v) BSA in PBS. Fixed cells were stained for caveolin, coronin-1C, or cortactin; mounted in Prolong®Antifade (Invitrogen); and photographed on a Leica SP5-II confocal laser-scanning microscope using a ×100, numerical aperture 1.4 PlanApo objective. Maximum projection images were compiled, bandpass-filtered, and analyzed using ImageJ software. Colocalization was quantified by measuring intensity profiles of caveolin and Coro1C across the lateral membrane or leading edge and calculated by Pearson correlation.

##### FRET Analysis of Rac Activity

Fibroblasts transfected with the Raichu Rac probe (18) and, where appropriate, pmCherry-caveolin-1 were filmed on 50K-coated MATTEK dishes for 10 min before stimulation with H/0 for 40 min. Images were acquired on a Leica DM IRBE inverted microscope using a ×63, numerical aperture 1.32 objective, Sutter DG5 light source, and Photometrics Coolsnap HQ2 camera, capturing images through CFP and YFP filters upon excitation through the CFP channel every 2 min. FRET ratios were calculated as described previously (19) using ImageJ software. Briefly, aligned CFP and YFP images were corrected for uneven illumination and photobleaching and background-subtracted. A binary mask was used to define the borders of the cell, and the YFP image was divided by the CFP image to yield a ratio image reflecting the distribution of Rac1 activity across the cell. The same result was obtained when the order of acquisition of CFP and YFP images was reversed or when fixed cells were analyzed, eliminating motion artifacts.

##### Photoactivatable GFP-Rac1

PAGFP-Rac1 and pmCherry were cotransfected into MEFs already transfected with control, Coro1C, or caveolin antisense oligos. Transfected cells were spread on fibronectin and identified by mCherry expression. A 2.3-μm^2^ spot at the non-protrusive membrane was photoactivated at 405 nm, and then GFP fluorescence at 488 nm was recorded at 2 images/s on an Ultraview spinning disk confocal microscope (PerkinElmer Life Sciences) using a ×63, numerical aperture 1.3 PlanApo objective. Images were analyzed using ImageJ and GraphPad Prism.

##### Migration Analysis

Cells were spread on CDM in medium with 10% serum for 4 h before capturing time-lapse images at 10-min intervals for 10 h on a Leica AS MDW microscope using a ×5, numerical aperture 0.15 Fluotar or ×40, numerical aperture 0.55 N PLAN objective and Roper charge-coupled device camera. The migration paths of all non-dividing, non-clustered cells were tracked using ImageJ software. The total absolute curvature of each track was calculated as described previously (6).

## Results

### 

#### 

##### Constitutive and Fibronectin-induced Mechanisms for Releasing Rac1 from the Membrane

**FIGURE 1.**
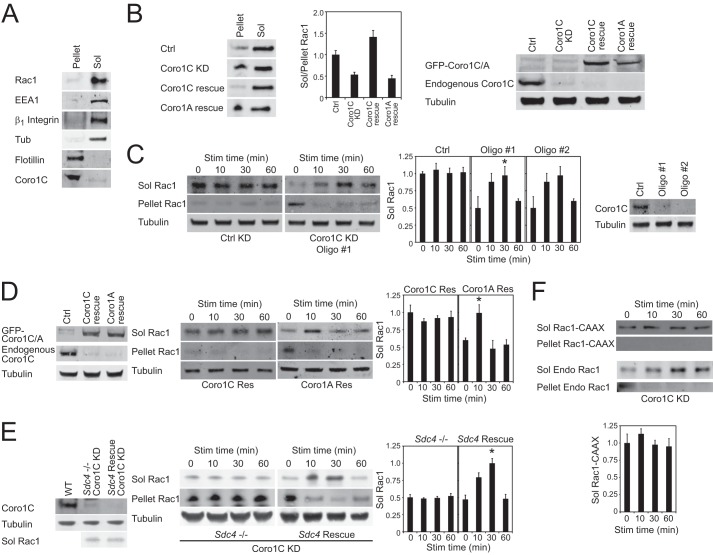
**Constitutive and inducible mechanisms for Rac1 mobilization.** Serum-starved fibroblasts spread on a minimal integrin-binding ligand (50K) were fractionated into detergent-soluble and -insoluble fractions and analyzed for protein segregation. *A*, separation of cells into a detergent (1% Nonidet P-40)-resistant membrane that includes flotillin-2 and Coro1C and detergent-soluble (*Sol*) lysate that includes Rac1, EEA1, β_1_-integrin, and tubulin (*Tub*) (*n* = 8). *B*, knockdown (*KD*) of Coro1C blocks the constitutive release of Rac1 from the membrane, causing accumulation in the detergent-resistant pellet. *Right panel*, efficient knockdown of Coro1C and re-expression of GFP-Coro1C and GFP-Coro1A to comparable levels. *Ctrl*, control. *C–F*, stimulation of Coro1C knockdown fibroblasts with fibronectin fragment H/0 reveals an alternative inducible redistribution mechanism. *C*, distribution of Rac1 between detergent-soluble and -insoluble fractions in control and Coro1C knockdown cells using two alternative oligos. *Right panel*, efficient knockdown of Coro1C. *Stim*, stimulation. *D*, exogenous Coro1C, but not the homologue Coro1A, rescues Rac1 localization in Coro1C knockdown cells. *Left panel*, efficient knockdown of endogenous Coro1C and equivalent rescue with coronin isoforms. *E*, distribution of Rac1 between detergent-soluble and -insoluble fractions upon Coro1C knockdown in syndecan-4 (*Sdc4*)-null and rescue cells. *Left panel*, efficient Coro1C knockdown and comparable levels of soluble Rac1 in unstimulated cells. *F*, localization of a non-prenylated C*AAX*-mutant Rac1 is unaffected by Coro1C expression, demonstrating the role of membrane association. Both endogenous (wild-type) and exogenous (C*AAX*-mutant) Rac1 are detected in the same experiment. *Error bars* indicate mean ± S.E. Significance was tested by analysis of variance. *, *p* < 0.05 (*n* = 4).

It has been established that fibronectin triggers activation of Rac1 (20, 21) and that this involves cooperative engagement of two separate fibronectin receptors, α_5_β_1_-integrin and syndecan-4 (17). However, the effect of fibronectin on Rac1 trafficking is less well studied. We reported recently that the actin-binding protein Coro1C redistributes GDP-Rac1 from the non-protrusive sides and rear of the cell (lateral membrane) to the leading edge for reactivation (6), but it is still unclear whether this is a constitutive pathway and whether additional trafficking mechanisms operate in response to fibronectin fragments. To investigate basal and fibronectin-stimulated trafficking, cells were treated with cycloheximide to prevent matrix synthesis and spread in serum-free medium on a 50-kDa fragment of fibronectin (50K) that encompasses the binding site for α_5_β_1_-integrin but not syndecan-4 (14). In control MEFs, Rac1 was detected in detergent-soluble (1% Nonidet P-40) lysate that comprised cytosol and soluble membrane, including β_1_-integrin and vesicle markers (Fig. 1, *A* and *B*). Coro1C was found predominantly in the detergent-resistant fraction that included flotillin microdomains. Knockdown of Coro1C shifted Rac1 from a detergent-soluble to a detergent-resistant membrane and could be rescued by re-expression of Coro1C but not Coro1A (Fig. 1*B*), demonstrating that expression of Coro1C is necessary for the maintenance of detergent-soluble Rac1 in cells spread on a minimal integrin-binding ligand.

To test the effect of engaging both fibronectin receptors, cells prespread on the integrin ligand (50K) were stimulated with a soluble syndecan-4-binding fragment of fibronectin comprising type III repeats 12–15 (H/0) (15). In control cells, where Rac1 resides in the detergent-soluble fraction, the distribution of Rac1 did not change (Fig. 1*C*). However, in Coro1C knockdown cells, the Rac1 trapped in the detergent-resistant pellet redistributed to the soluble fraction upon H/0 stimulation. Wild-type behavior could be restored by re-expression of Coro1C but not Coro1A (Fig. 1*D*). To pinpoint the receptor responsible for H/0-stimulated redistribution, Coro1C was knocked down in syndecan-4-null and syndecan-4-rescued MEFs. Coro1C knockdown in either syndecan-4-null or rescue cells resulted in similar levels of detergent-soluble Rac1 in unstimulated cells (Fig. 1*E*). However, unlike the rescue cells, syndecan-4-null cells failed to respond to H/0, demonstrating that the inducible redistribution is syndecan-4-dependent. To test whether partitioning of Rac1 between the soluble and insoluble fractions was due to membrane association, GFP-Rac1 with a cysteine-to-serine substitution in the C*AAX* box was transfected into Coro1C-knockdown cells. Rac1-C*AAX* remained in the soluble fraction and did not redistribute upon syndecan-4-engagement, although the distribution of endogenous Rac1 in the same cells was still affected by Coro1C knockdown (Fig. 1*F*). Together, these experiments demonstrate that Coro1C regulates the constitutive release of Rac1 from the detergent-insoluble membrane but that a second, inducible mechanism can release Rac1 upon engagement of syndecan-4 by fibronectin.

##### Caveolin and Coronin-1C Provide Parallel Rac1 Release Mechanisms

**FIGURE 2.**
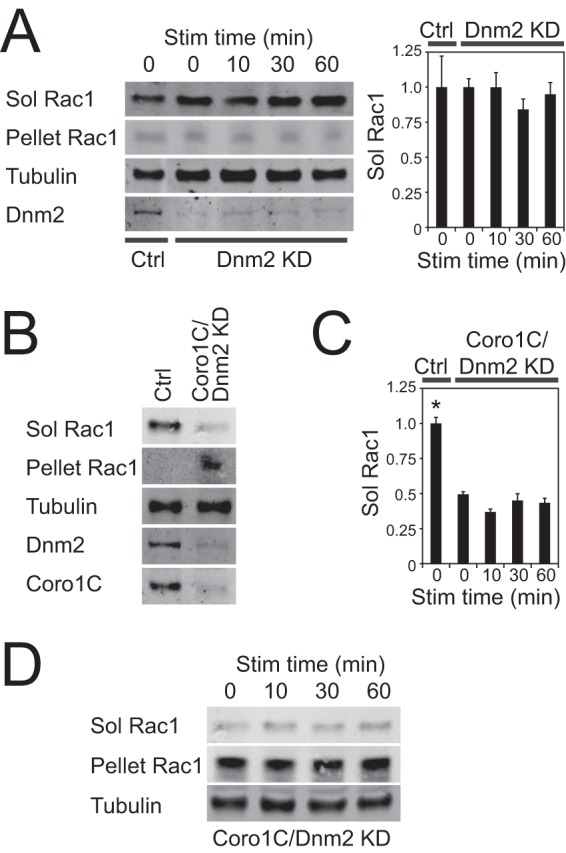
**Dynamin dependence of Rac1 trafficking.** Serum-starved fibroblasts spread on 50K and stimulated with H/0 were fractionated into detergent-soluble and -insoluble fractions. *A*, Rac1 remains in the soluble (*Sol*) fraction when dynamin-2 (*Dnm2*) is knocked down (*KD*) to 7.4% ± 3.2% of normal. *Ctrl*, control; *Stim*, stimulation. *B–D*, Rac1 is retained in the detergent-resistant fraction (*B* and *C*) when Coro1C and dynamin-2 are knocked down to 7.1% ± 5.0% and 6.9% ± 2.8%, respectively, and fail to redistribute in response to fibronectin (*C* and *D*). *Error bars* indicate mean ± S.E. *, *p* < 0.05 (*n* = 4). *B–D* are from experiments run in parallel.

Redistribution of membrane-associated proteins is frequently achieved through dynamin-dependent endocytosis. To test whether entrapment of Rac1 in the detergent-resistant membrane of Coro1C-depleted cells was a consequence of defective dynamin-dependent endocytosis, we tested whether dynamin-2 knockdown affected the segregation of Rac1. As in control cells, Rac1 remained in the soluble fraction in dynamin-2 knockdown cells and was unaffected by engagement of syndecan-4 (Fig. 2*A*). Furthermore, knockdown of Coro1C still shifted Rac1 from the soluble fraction to the detergent-resistant pellet in dynamin-2-depleted cells (Fig. 2, *B* and *C*), demonstrating that the effect of Coro1C is dynamin-independent. However, Coro1C/dynamin-2 double knockdown did prevent the redistribution of Rac1 to the soluble fraction upon syndecan-4 engagement (Fig. 2, *C* and *D*), demonstrating that the alternative pathway involves dynamin-dependent endocytosis.

**FIGURE 3.**
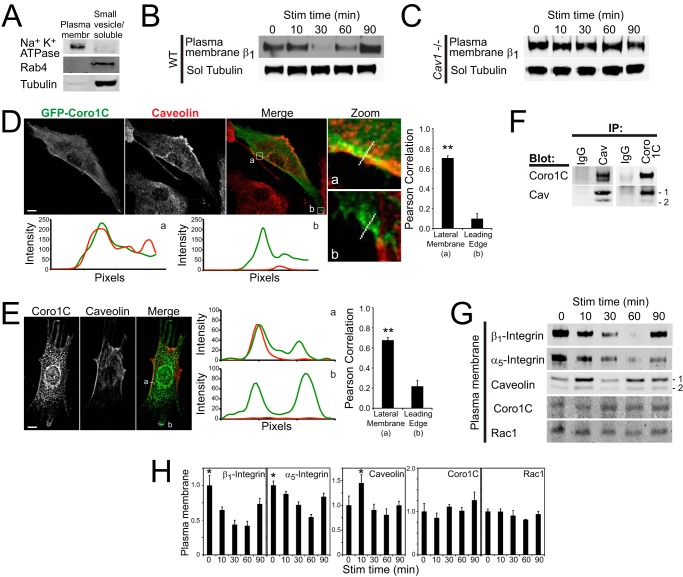
**Caveolin and Coro1C colocalize in the lateral membrane.** *A*, mechanical fractionation of fibroblasts into total plasma membrane (*membr*) (ATPase) and soluble/small vesicle (Rab4 + tubulin) fractions. *B* and *C*, H/0 stimulation of wild-type (*B*) but not caveolin knockout (*C*) fibroblasts triggers integrin endocytosis (*n* = 4). *Sol*, soluble; *Stim*, stimulation. *D* and *E*, caveolin colocalizes with GFP-Coro1C (*D*) and endogenous Coro1C (*E*) in the lateral membrane of fibroblasts spread on fibronectin. Images were acquired on a confocal laser-scanning microscope and are representative of 60 images from three separate experiments. Intensity plots indicate the pixel intensity across the lateral membrane (*a*) and lamella membrane (*b*) of each representative image, and graphs represent the average Pearson correlation in each region. *Scale bars* = 10 μm. *F*, caveolin (*Cav*) and Coro1C coimmunoprecipitate (*IP*) from the non-clarified lysates of sonicated fibroblasts (*n* = 5). Bands for caveolin-1 and 2 are indicated. *G* and *H*, plasma membrane fractions of wild-type fibroblasts, prepared as in *A*, were blotted for integrin, caveolin, Coro1C, and Rac1. *G*, representative Western blot analysis. *H*, average of four experiments. *Error bars* indicate mean ± S.E. *, *p* < 0.05; **, *p* < 0.005.

It has been reported that Rac1 is subject to caveolin-dependent endocytosis (12, 13), and we have reported previously that syndecan-4 engagement triggers caveolin-dependent endocytosis of α_5_β_1_-integrin (10). To test the dynamics of α_5_β_1_-integrin redistribution in response to syndecan-4-engagement, the total plasma membrane (including Na^+^ K^+^ ATPase) was separated from the soluble/small vesicle components of the cell (including Rab4 and tubulin) by rupturing the cell by sonication and harvesting large membranes by centrifugation (Fig. 3*A*). The minima in plasma membrane-associated α_5_β_1_-integrin at 30 min (Fig. 3*B*) correlated with the peak in cytosolic Rac1 in Coro1C knockdown cells (Fig. 1*C*). Integrin redistribution could be blocked by knockout of caveolin-1 (Fig. 3*C*), causing us to examine the role of caveolin in the movement of Rac1.

Coro1C localizes to both protruding membrane tips and the non-protruding membrane at the back and sides of the cell in proximity to actin stress fibers (from here on termed the lateral membrane) (6, 22). Caveolin colocalized with GFP-tagged (Fig. 3*D*) or immunostained (Fig. 3*E*) Coro1C in the lateral membrane (Fig. 3, *D* and *E*, *a*) but not at the leading edge (Fig. 3, *D* and *E*, *b*), supporting the hypothesis that Coro1C and caveolin might provide alternative means to remove Rac1 from the lateral membrane. Retention of Coro1C and a proportion of caveolin in the detergent-resistant pellet of cell lysates prevented analysis of association by conventional coimmunoprecipitation. However, caveolin and Coro1C could be coimmunoprecipitated from the non-clarified lysates of sonicated fibroblasts using magnetic beads, demonstrating that the proteins are associated with shared membrane fragments (Fig. 3*F*). The relationship between caveolin-mediated integrin endocytosis and the localization of Coro1C and Rac1 was investigated by further analysis of the plasma membrane fraction of fibroblasts lysed by sonication. As reported previously (10), within 10 min of syndecan-4-engagement, caveolin was recruited to the plasma before redistribution away from the plasma membrane over a similar time period to both α_5_- and β_1_-integrin (Fig. 3, *G* and *H*). Importantly, levels of plasma membrane-associated Coro1C and Rac1 were unaffected by syndecan-4 engagement (Fig. 3, *G* and *H*), consistent with the detergent fractionation data (Fig. 1*C*). Therefore, although caveolin and Coro1C mediate the release of Rac1 from a similar membrane compartment (Fig. 3, *D–F*), they do not do so as part of a single trafficking complex (Fig. 3, *G* and *H*), leading us to look for differences between these superficially redundant pathways.

**FIGURE 4.**
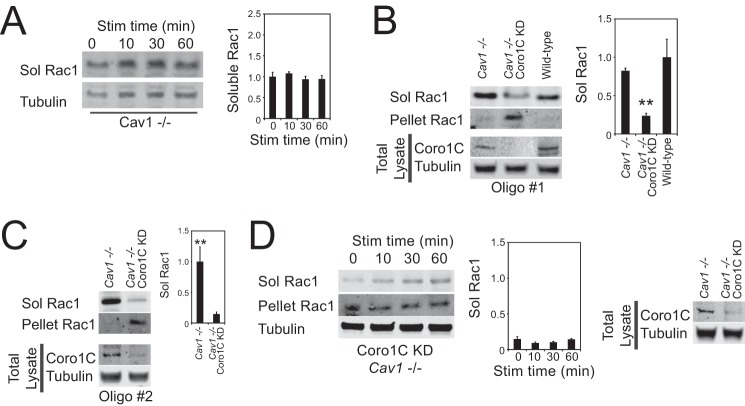
**Caveolin mediates the fibronectin-dependent release of Rac1 from the lateral membrane.** Serum-starved fibroblasts spread on a minimal integrin-binding ligand (50K) were fractionated into detergent-soluble and -insoluble fractions and analyzed for protein segregation. *A*, H/0 stimulation has no effect on the distribution of Rac1 between fractions in caveolin-null MEFs. *Sol*, soluble; *Stim*, stimulation. *B* and *C*, knockdown of Coro1C with two alternative oligos but not caveolin blocks the constitutive release of Rac1 from the membrane, causing accumulation in the detergent-resistant pellet. *D*, knockdown of caveolin blocks the fibronectin-induced release of Rac1 from the membrane of Coro1C-depleted fibroblasts. *Error bars* indicate mean ± S.E. Significance was tested by analysis of variance. **, *p* < 0.005 (*n* = 6).

Knockout or knockdown of caveolin-1 causes concomitant loss of caveolin-2, thereby blocking caveolin-dependent endocytosis (23). In *Cav1*^−/−^ MEFS, where Coro1C-dependent trafficking was intact, Rac1 remained in the detergent-soluble fraction and was unaffected by H/0 (Fig. 4, *A* and *B*), similar to dynamin-2 knockdown fibroblasts. Depletion of Coro1C caused Rac1 to accumulate in the detergent-resistant fraction in *Cav1*^−/−^ MEFs in the absence of syndecan-4 ligand (Fig. 4, *B* and *C*), just as Coro1C knockdown did in wild-type MEFs (Fig. 1*B*). However, engagement of syndecan-4 did not cause Rac1 to redistribute in Coro1C knockdown *Cav1*^−/−^ cells (Fig. 4*D*), demonstrating that the fibronectin-triggered mechanism for Rac1 redistribution is mediated by caveolin.

**FIGURE 5.**
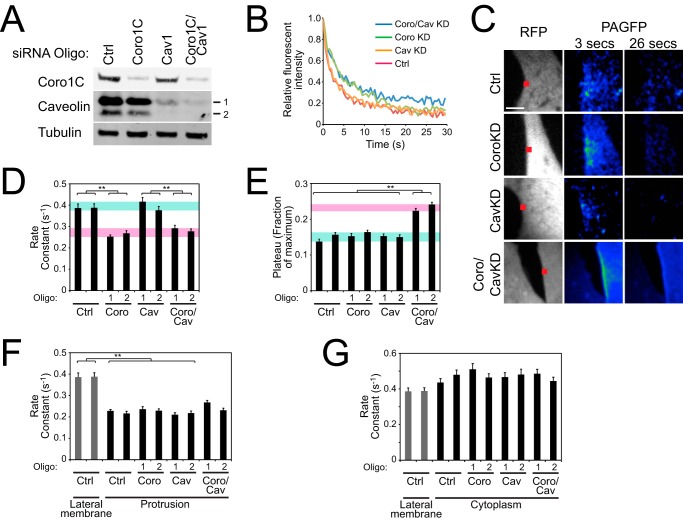
**Coro1C and caveolin have alternative effects on the dynamics of Rac1 release.** Fibroblasts expressing photoactivatable GFP-tagged Rac1 were spread on fibronectin, and the fluorophore was activated at 405 nm at a 1.5-μm spot on the lateral membrane before measuring the dissipation of the fluorescent signal at 488/525 nm. *A*, knockdown of Coro1C and caveolin in analyzed cells. *Ctrl*, control. *B*, fluorescence intensity profiles at the point of photoactivation. *Coro*, Coro1C; *Cav*, caveolin; *KD*, knockdown. *C*, cotransfected RFP images to indicate membrane boundaries and area of photoactivation (*red squares*) and example fluorescent images 3 s after photoactivation. Images were taken from supplemental Movie S1. *D* and *E*, rate constants (*D*) and plateaus (*E*) of fluorescent decay calculated by fitting one-phase decay curves. *Green* and *red bands* indicate groups of “mobile” or “retarded” data, respectively. *F* and *G*, rate constants of fluorescent decay at the protrusive membrane (*F*) and in the cytoplasm (*G*) in comparison with control values at the lateral membrane. *Error bars* indicate mean ± S.E. Significance was tested by F-test. **, *p* < 0.005 (*n* = 18).

To test the model further, we examined the diffusion of photoactivatable GFP-tagged Rac1 from the lateral membrane. We have reported previously that dispersion of Rac1 from the lateral membrane is retarded by knockdown of Coro1C (6), and so we tested whether Caveolin knockdown had a similar effect. Following photoactivation of a 1.5-μm square of lateral membrane, fluorescence quickly dispersed in control cells (*t*_½_ = 1.79 s). Coro1C knockdown retarded diffusion (*t*_½_ = 2.58 s), but caveolin knockdown had no effect (*t*_½_ = 1.84 s) (Fig. 5, *A–D*, and supplemental Movie S1). Knockdown of both Coro1C and caveolin had no effect on the diffusion rate constant beyond the effect observed in Coro1C knockdown cells (*t*_½_ = 2.50 s) but resulted in a higher fluorescent plateau (Fig. 5, *B* and *E*, and supplemental Movie S1), demonstrating that, in the absence of both Coro1C and caveolin, a proportion of Rac1 is trapped irreversibly at the lateral membrane. Unlike release from the lateral membrane, diffusion of GFP-Rac1 from the protrusive membrane was slow and independent of Coro1C and caveolin (Fig. 5*F*), demonstrating that Coro1C and caveolin traffic Rac1 from specific regions of the membrane. Free diffusion of cytosolic Rac1 was fast and independent of Coro1C and caveolin (Fig. 5*G*). Collectively these experiments show that Coro1C and caveolin mediate the release of Rac1 from the lateral membrane by separate pathways and that Coro1C forms the primary (constitutive) mechanism because the effects of caveolin are only detected in the absence of Coro1C.

##### In the Absence of Coro1C, Caveolin Targets Rac1 toward Proteasomal Degradation

**FIGURE 6.**
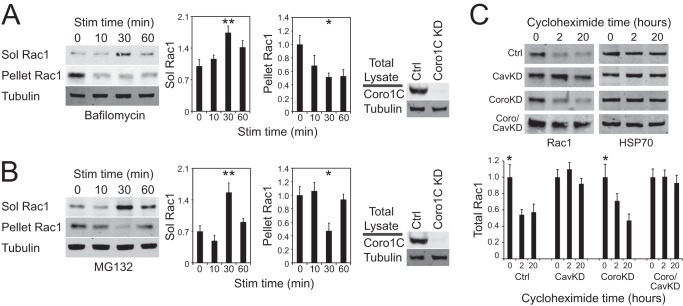
**Effects of trafficking on Rac1 degradation.** *A* and *B*, serum-starved fibroblasts treated with the lysosomal inhibitor bafilomycin (*A*) or the proteasomal inhibitor MG132 (*B*) were spread on 50K, stimulated with H/0, and fractionated into detergent-soluble (*Sol*) and -insoluble fractions. Images represent the distribution of Rac1 between detergent-soluble and -insoluble fractions and validation of Coro1C knockdown. *Stim*, stimulated; *Ctrl*, control. *C*, the inhibitor of protein synthesis cycloheximide was added to cells in culture and lysates made over time to detect the degradation of Rac1. Validation of the knockdown (*KD*) is shown in Fig. 5*A. Error bars* indicate mean ± S.E. Significance was tested by analysis of variance. *, *p* < 0.05; **, *p* < 0.005 (*n* = 5).

It has been reported that Rac1 is subject to proteasomal degradation (24, 25) and also that caveolar endocytosis of Rac1 leads to degradation (13). It is noticeable that, when Coro1C knockdown cells were stimulated with H/0, Rac1 moved from pellet to soluble fractions at 30 min but disappeared from the soluble fraction at 60 min without returning to the pellet (Fig. 1*C*). We therefore examined the effect of protein degradation on Rac1 distribution. The lysosomal inhibitor bafilomycin had no effect on Rac1 redistribution from pellet to soluble fractions or the subsequent disappearance from both fractions in stimulated Coro1C knockdown cells (Fig. 6*A*). By contrast, inhibition of the proteasome with MG132 still allowed movement to the soluble fraction, but Rac1 subsequently returned to the detergent-resistant fraction rather than being degraded (Fig. 6*B*). The differing effects of Coro1C and caveolin-dependent trafficking on Rac1 degradation were also demonstrated by treating cells in culture with cycloheximide to prevent the synthesis of new protein. In cycloheximide-treated control cells, Rac1 degradation was detected by the fall in protein levels over 20 h, but inhibition of the caveolin trafficking pathway using siRNA blocked Rac1 depletion (Fig. 6*C*). Knockdown of Coro1C did not block the disappearance of Rac1, confirming that caveolin and Coro1C do not form a single pathway and demonstrating that Coro1C is not a major contributor to degradative Rac1 trafficking. Knockdown of both caveolin and Coro1C prevented Rac1 depletion by blocking the caveolin pathway. Together, these experiments demonstrate that, although Coro1C provides the more efficient mechanism for releasing Rac1 from the plasma membrane, the caveolin release mechanism reported previously provides an important alternative pathway that traffics Rac1 toward degradation.

##### Caveolin Retards Activation of Rac1

**FIGURE 7.**
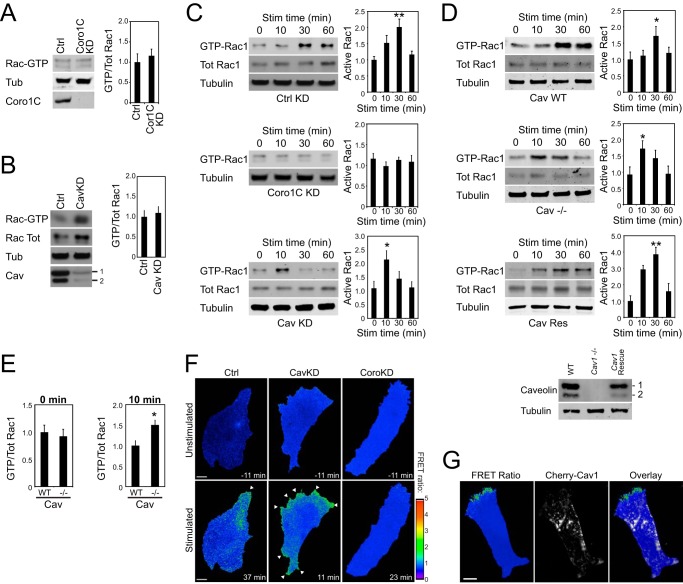
**Caveolin retards Rac1 activation by defining inactive membrane regions.** Serum-starved fibroblasts spread on a 50K and stimulated with H/0 were assayed for Rac1 activation by effector pulldown. *A*, unstimulated control (*Ctrl*) and Coro1C knockdown (*KD*) MEFs. *Tub*, tubulin. *B*, unstimulated control and caveolin (*Cav*) knockdown MEFs. *C*, control, Coro1C, and caveolin knockdown MEFs stimulated (*Stim*) with H/0. *D* and E, wild-type, caveolin knockout, and rescued caveolin knockout MEFs (*D*), including pairwise comparison at 0 and 10 min of stimulation (*E*). *F* and *G*, analysis of Rac1 activity distribution using a Raichu-Rac FRET probe. *F*, fibroblasts spread on 50K before or after H/0-stimulation. Images were taken from supplemental Movie S2 at the peak of the Rac1 response, as determined by biochemical assays. *G*, fibroblasts coexpressing pmCherry-caveolin spread on fibronectin and fixed. Results are representative of at least six separate experiments. *Error bars* indicate mean ± S.E. Significance was tested by analysis of variance. *, *p* < 0.05; **, *p* < 0.005. *Scale bars* = 10 μm.

In this work, we demonstrated that Coro1C and caveolin play independent roles in the removal of Rac1 from the lateral membrane. Although Coro1C redistributes Rac1 from the lateral to the protruding membrane and is necessary for activation in response to the H/0 fibronectin fragment (Figs. 1 and 5 and Ref. 6), the caveolin-dependent mechanism targets Rac1 for degradation (Fig. 6) so that Coro1C and caveolin could be broadly classified as positive and negative regulators of Rac1, respectively. This hypothesis led us to compare the effects of Coro1C and caveolin on the dynamics of Rac1 activation. In cells spread on the minimal 50K ligand, knockdown of Coro1C or caveolin did not affect the proportion of active Rac1 (Fig. 7, *A* and *B*), although knockdown of caveolin did increase the amount of total Rac1 in cells, as reported previously (12, 13) and consistent with the role of caveolin in Rac1 degradation (Fig. 6). It was not until H/0-induced Rac1 activation was examined that the difference between Coro1C and caveolin manifested. Unlike control cells, Coro1C knockdown cells did not activate Rac1 in response to H/0, establishing the role of Coro1C as a positive regulator of Rac1 (Fig. 7*C*). By contrast, Rac1 was activated in caveolin knockdown cells, but, interestingly, activation was accelerated in knockdown cells (Fig. 7*C*), consistent with the negative role of caveolin. Likewise, MEFs from *Cav1*^−/−^ mice exhibited accelerated Rac1 activation that included a 50% increase in activity at 10 min (Fig. 7, *D* and *E*). Normal kinetics could be rescued by re-expression of caveolin-1 (Fig. 7*D*). The difference in kinetics was not due to altered syndecan-4 expression, which was comparable between cell lines (data not shown). Together, these experiments demonstrate that caveolin affects the kinetics of Rac1 activation and are supported by previous reports that membrane association of Rac1 is increased and mislocalized in *Cav1*^−/−^ MEFs (26).

We hypothesized that the accelerated Rac1 activation in cells lacking caveolin is due to widespread activation compared with localized activation in caveolin-expressing cells. MEFs transfected with the Raichu-Rac biosensor were spread on 50K and stimulated with soluble H/0 to trigger Rac1 activation in a non-localized fashion. Despite the diffuse nature of the stimulus, Rac1 activation was polarized toward a dominant protrusion in control fibroblasts, indicating that the cells already possessed intrinsic polarity (Fig. 7*F* and supplemental Movie S2). By contrast, Rac1 was activated around the entire periphery of caveolin knockdown fibroblasts as well being accelerated, demonstrating that caveolin defines regions where Rac1 cannot be activated. Consistent with the biochemical data, knockdown of Coro1C prevented activation of Rac1 altogether and re-emphasized the difference between the roles of Coro1C and caveolin. The spatial segregation of active Rac1 from caveolin was investigated by cotransfection of mCherry-caveolin and the biosensor into caveolin-depleted fibroblasts. As well as vesicles, mCherry-caveolin localized to non-protrusive edges of cells, defining regions where Rac1 could not be activated, restoring polarized Rac1 activation (Fig. 7*G*). Together, these experiments demonstrate that caveolin defines regions of the membrane where Rac1 is not activated, even in the presence of a matrix stimulus. This arrangement confers intrinsic polarity on cells by defining regions where membrane protrusion is possible even before it occurs.

##### Caveolin-dependent Polarity Is Required for Processive Cell Migration

**FIGURE 8.**
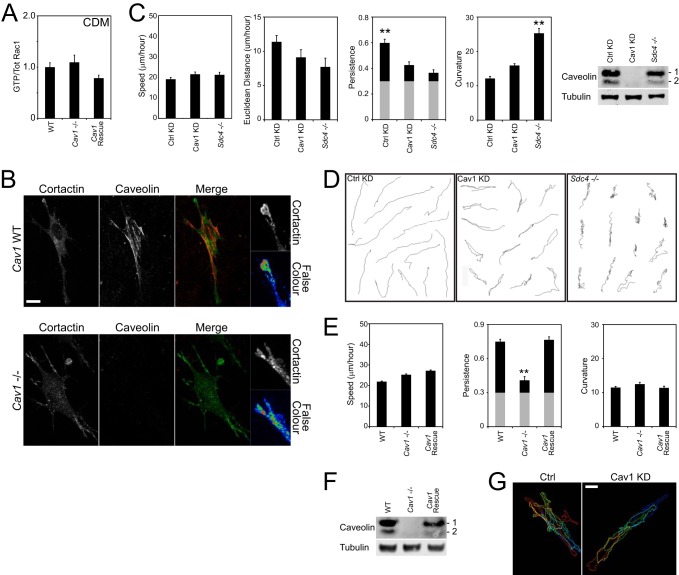
**Caveolin is necessary for the localization of membrane protrusion in a fibrillar matrix.** Fibroblasts were allowed to embed into cell-derived matrix for 4 h before preparing lysates for Rac1 activity, fixing, or tracking migration for 10 h. *A*, measurement of Rac1 activity in a fibrous environment by effector pulldown. *B*, distribution of cortactin and caveolin in CDM. False color images represent the intensity of cortactin staining. *C* and *E*, values of speed (distance/time), Euclidean distance per hour (displacement/time), persistence (displacement/distance), and curvature (see “Experimental Procedures” and Ref. 6) were calculated for each cell. *Gray columns* indicate the experimentally determined threshold for random migration. *C* and *F*, Western blots indicating the efficiency of caveolin knockdown or re-expression in migration experiments as appropriate. The results represent an analysis of >100 cells/condition. *Error bars* indicate mean ± S.E. Significance was tested by Kruskal-Wallis test. **, *p* < 0.005. *D*, example migration tracks from *C. G*, time projection of the outlines of β_1_-integrin-GFP fibroblasts at 2.5-h intervals. Images were derived from supplemental Movie S4. *Scale bars* = 10 μm.

The effect of caveolin on Rac1 polarization led us to examine the effect on fibroblast migration through a fibrous matrix. It has been reported that caveolin expression affects both the organization of secreted extracellular matrix and the persistence of migration of the individual cells (12, 26), but a more in-depth analysis of migration is required if we are to compare the different mechanisms of Rac1 regulation. We recently demonstrated that preventing Rac1 activation by either knocking down Coro1C or Rac1 itself had no effect on the ability of cells to migrate along a fibronectin fiber but caused cells to “forget” the direction of migration and, therefore, shunt backward and forward (6). At the same time, we demonstrated that mislocalization of active Rac1 by knockdown of the sequestering molecule, RCC2, also induced shunting behavior because of the formation of multiple active Rac1 protrusions. Therefore, we examined the migration of cells lacking caveolin on fibrous matrix generated by wild-type fibroblasts to eliminate contributions from an altered matrix structure. As on 2D fibronectin, caveolin expression had no effect on the proportion of Rac1 that was active in cells on cell-derived matrix (CDM) (Fig. 8*A*). Cortactin was used as a reporter of a protrusive membrane because autofluorescent components of the CDM compromise the veracity of the FRET analysis. Unlike wild-type cells, where cortactin is focused at the tips of one or two protrusions, cortactin was distributed along the length of a number of membrane extensions in caveolin knockout cells (Fig. 8*B*). Neither knockdown of caveolin nor knockout of syndecan-4, the Rac1-trigger, had a reproducible effect on migration speed but caused a dramatic reduction in Euclidean distance per hour and linear persistence (Euclidean distance / total distance) of migration, which suggested more random migration (Fig. 8*C*). However, inspection of the migration paths revealed that the modes of migration were very different. The migration paths of *Sdc4*^−/−^ MEFs frequently included regions of high curvature and direction change because of constitutively high Rac1 activity that was unaffected by fibronectin engagement (Fig. 8*D* and Ref. 17). By contrast, Cav1-depleted MEFs have normal average Rac1 activity and respond to fibronectin but fail to localize the signal. Therefore, Cav1-depleted MEFs moved linearly along matrix fibers like a wild-type cell but, because of the absence of a dominant protrusion, shunted backward and forward on the fibers and exhibited reduced persistence, like a *Sdc4*^−/−^ cell (Fig. 8*D* and supplemental Movie S3). Linear processive migration, high-curvature migration, and shunting migration were quantified by calculating the curvature of each track as described previously (6). The high-curvature migration of *Sdc4*^−/−^ resulted in a curvature value that was double that of control MEFs (Fig. 8*C*). By contrast, the curvature of caveolin-depleted MEFs was only slightly higher than that of control MEFs despite the compromised persistence. The shunting behavior indicated that caveolin-depleted MEFs were still capable of recognizing the fiber and following a linear trajectory, albeit in a non-persistent fashion, and was consistent with the presence of active Rac1 protrusions at either end of the cell. Similar results were obtained with knockout MEFs. Knockout of caveolin 1 had little effect on curvature but lowered persistence and could be rescued by re-expression of exogenous caveolin (Fig. 8, *E* and *F*). To ensure that shunting was a protrusion rather than a tail-retraction defect, caveolin was depleted in MEFs stably expressing β_1_-integrin-GFP, which allowed the cell boundaries to be clearly visualized. Although retraction fibers were seen in both control and knockdown cells, the direction changes of caveolin-depleted cells occurred when a different protrusion achieved dominance and were not due to a detachment defect (Fig. 8*G* and supplemental Movie S4).

Collectively, these experiments demonstrate that regulating the localization of active Rac1 is as important as regulating activity itself. Although shunting migration is more constrained than entirely random migration, in a three-dimensional environment, the net effect on cell displacement is similar. Furthermore, we found that influences beyond the traditional mechanisms of guanine-nucleotide exchange factor-mediated activation play critical roles and that competing trafficking pathways, through coronin-1C and caveolin, have important effects on cell behavior.

## Discussion

In this work, we discover that Coro1C and caveolin provide alternative mechanisms for removing Rac1 from lateral membrane; that Coro1C-mediated redistribution is constitutive and required for Rac1 activation, whereas caveolin-dependent endocytosis drives Rac1 degradation in response to fibronectin; and that the negative effect of caveolin on Rac1 defines regions of the membrane that are resistant to protrusion formation and results in processive, linear migration.

**FIGURE 9.**
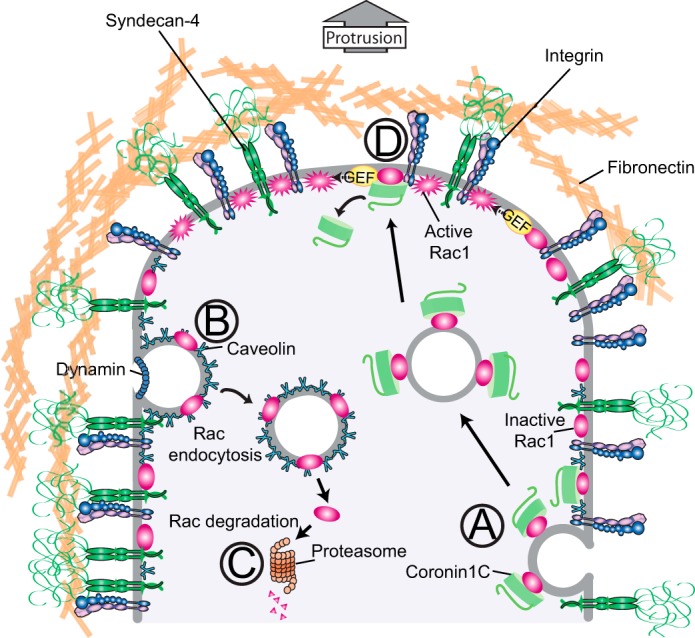
**Schematic of the potential fates of Rac1 in a migrating cell.** In the absence of fibronectin, Coro1C redistributes Rac1 (*A*). During established migration, the presentation of fibronectin at the caveolin-rich sides and rear of the cell triggers Rac1 endocytosis (*B*) and degradation (*C*). At the front of the cell, where caveolin levels are low, fibronectin triggers guanine-nucleotide exchange factor-mediated Rac1 activation (*D*). The role of caveolin in Rac1 trafficking complements the role of caveolin in fibronectin receptor trafficking, as summarized by Caswell *et al.* (37).

The critical difference between Coro1C and caveolin mechanisms is fibronectin dependence, which, for the first time, provides an explanation of how cells maintain polarity for the long period of time over which wound healing occurs. In unwounded skin, dermal fibroblasts are quiescent and distributed diffusely. Wounding immediately introduces chemotactic signals that include fibronectin leaking from damaged vessels, PDGF secreted from platelets as they plug the vessels, and further fibronectin and growth factors secreted from recruited leukocytes (27). Under these circumstances, it is advantageous for each fibroblast to be able to initiate migration in the direction of the chemotactic gradient. The absence of fibronectin from unwounded dermis means that the caveolin pathway is not active, so Rac1 will be available across the membrane and redistributed by Coro1C (Fig. 9*A*). The problem with the chemotactic gradient model is that fibronectin is quickly incorporated into the fibrous, collagen-rich matrix so that the gradient is transient, and, as the cell migrates, it quickly becomes surrounded by fibronectin. The trafficking mechanisms that we distinguish in this work, particularly the role of caveolin, provide a mechanism for a cell to remember the direction of the gradient even after it has faded. Polarization of cells, upon initial exposure to a chemoattractant, positions caveolin at the rear and sides of the cell, away from the leading lamella. Over time fibronectin is incorporated into the dermal matrix, so the cell becomes sounded in fibronectin and the subcellular signaling depends on the arrangement of membrane structures rather than the external gradient. At the caveolin-rich membrane at the cell rear, fibronectin stimulates the removal and degradation of Rac1 (Fig. 9, *B* and *C*). At the front of the cell, in the absence of caveolin, Rac1 activation signals dominate and sustain forward migration (Fig. 9*D*).

There are a number of pieces of evidence to support this model. Our CDM matrix migration experiments involve seeding cells onto fibers that are rich in both collagen and fibronectin and lack polarity (17). If fibronectin drives protrusion only, then these cells should shunt backward and forward as different protrusions achieve dominance. In fact, wild-type cells migrate in straight lines, and it is only in the absence of caveolin that they shunt, demonstrating the importance of caveolin in maintaining polarity (Fig. 7). The second demonstration of intrinsic polarity comes from FRET experiments (Fig. 7, *E* and *F*). In these experiments, cells are stimulated with diffuse soluble ligand. However, wild-type cells reproducibly activate Rac1 in a polarized fashion, demonstrating that polarity can be dictated by existing molecular arrangements within the cell and is not wholly dependent on an external gradient. Finally, it has been demonstrated that caveolin knockout mice do indeed suffer a wound healing delay because of defective fibroblast migration, confirming that the *in vitro* hypothesis is apparent *in vivo* (12).

The model is further supported by existing links between the engagement of syndecan-4 by fibronectin and caveolin-mediated endocytosis. We have reported previously that ligation of syndecan-4 causes activation of RhoG (10), and we and others have demonstrated that RhoG activation triggers caveolar endocytosis of integrins (10, 28). The wound healing defects of *Cav1*^−/−^ are mirrored by comparable healing delays in *Sdc4*^−/−^ and *Rhog*^−/−^ mice, demonstrating the functional synergy between the components of the pathway (8, 10). Furthermore, it has been reported that FGF2 activates RhoG, via syndecan-4, in a similar way as the fibronectin response we report here (29). It was found that polarized Rac1 activation by FGF2 could be perturbed by knockdown of RhoG, indicating that polarization of signals by caveolin-mediated trafficking could apply equally to the fibronectin and growth factor-stimulated signals that occur upon wounding.

An important question now is how the functions of Coro1C and caveolin are integrated with the effects of other coronin family members that have been found to influence trafficking and activation of Rac1. Coronin-1A binds to Rac1 through a cryptic CRIB domain, mediating the release of Rac1 from sequestration by RhoGDI and activation by β-Pix (ArhGEF7) (30, 31). Our unsuccessful attempts to rescue Coro1C knockdown with Coro1A (Fig. 1, *B* and *D*), combined with our previous demonstration that Coro1C does not affect the association of Rac1 with RhoGDI (6), means that Coro1C and 1A are not redundant. In the same way, it has been demonstrated that Coro1A and Coro1B are not redundant (32), so, although each of the type I coronins affects the same process, they each have a specific role. Indeed, Coro1C and Coro1A have directly antagonistic effects, promoting the release or recruitment of Rac1 to the plasma membrane, respectively. The most direct demonstration of the importance of coronins in Rac1 trafficking, rather than activation, is the observation that constitutively active Rac1 does not induce ruffles in COS1 cells depleted of Coro1A (32). The contrasting roles of Coro1C and Coro1A might suggest that coronin isoforms act as sequential cargo adapters as part of a trafficking pathway, and it is interesting to note that coronins act as trimeric complexes, although there is no evidence of heterotrimer formation so far.

The relationships between different coronins vary between cell types. Coro1A is not ubiquitously expressed and found predominantly in hematopoietic cells. Although Coro1C is highly expressed in immune cells, the expression pattern is much broader and includes the fibroblasts that are the focus of this study. The effects of both Coro1C and Coro1A on Rac1 trafficking and actin organization are also manifested at a functional level, with both promoting cell migration. Coro1C expression has been found to be elevated in hepatocellular carcinoma and malignant glioblastoma, and expression has been found to correlate with motility in a number of cancer cell lines (33), whereas knockdown of Coro1C has a negative effect on fibroblast migration (6, 34). Likewise, the T cells of Coro1A knockout mice exhibit migration defects (35). The extent to which these defects are linked to GTPase trafficking *versus* regulation of the actin cytoskeleton through direct association with Arp2/3 requires further investigation.

The multifaceted nature of Rac1 activation will have consequences for how wound therapy is approached. PDGF, FGF, and VEGF have all been investigated as potential wound therapies, and, indeed, PDGF has Food and Drug Administration approval for use on human chronic wound patients (36). However, the approaches have met with mixed success, and the polarization events we identify here could explain those limitations. If poor healing is due to compromised growth factor or fibronectin secretion, the exogenous application of growth factor would be a successful therapy. However, if poor healing is due to compromised trafficking and polarization in fibroblasts, simply stimulating fibroblasts will not achieve the desired, long-range migration response. Therefore, it is important to understand the different aspects of Rac1 regulation if we are to stratify clinical patients and apply the appropriate treatment.

## Supplementary Material

Supplemental Data

## Footnotes

2 The abbreviations used are:

GDI: guanine-nucleotide dissociation inhibitor
CDM: cell-derived matrix
MEF: mouse embryonic fibroblast
DME: Dulbecco's modified Eagle's medium
CFP: cyan fluorescent protein
YFP: yellow fluorescent protein.
