# Supplementary material for: Coronin-1C Protein and Caveolin Protein Provide Constitutive and Inducible Mechanisms of Rac1 Protein Trafficking
Source: J Biol Chem. 2015 Apr 29;290(25):15437–49. doi: 10.1074/jbc.M115.640367 (PMC4505459; doi:10.1074/jbc.M115.640367)
Supplement: Supplemental Data [file supp_290_25_15437__index.html]

Coronin-1C and Caveolin Provide Constitutive and Inducible Mechanisms of Rac1 Trafficking — Coronin-1C Protein and Caveolin Protein Provide Constitutive and Inducible Mechanisms of Rac1 Protein Trafficking — Parallel Coro1C and Caveolin Rac1 Trafficking Mechanisms — Supplemental Data 

# Coronin-1C Protein and Caveolin Protein Provide Constitutive and Inducible Mechanisms of Rac1 Protein Trafficking

## Supplemental Data

**Files in this Data Supplement:**

- Movie Legends (.docx, 72 KB) - Movie Legends
- Movie S1 (.mov, 857 KB) - Movie S1
- Movie S2 (.mov, 990 KB) - Movie S2
- Movie S3 (.mov, 4.8 MB) - Movie S3
- Movie S4 (.mov, 2.7 MB) - Movie S4
