# Supplementary material for: Coronin-1C Protein and Caveolin Protein Provide Constitutive and Inducible Mechanisms of Rac1 Protein Trafficking
Source: J Biol Chem. 2015 Apr 29;290(25):15437–49. doi: 10.1074/jbc.M115.640367 (PMC4505459; doi:10.1074/jbc.M115.640367)
Supplement: Supplemental Data [file supp_M115.640367_jbc.M115.640367-1.docx]

**Supplementary Material**

**Movie S1. Coro1C and caveolin have alternative effects on the release of photoactivated GFP-tagged Rac1 from the lateral membrane.** PAGFP was photoactivated in a 1.5x1.5 μm box at the lateral edge of cells spread on fibronectin and release of Rac1 followed by decay of GFP fluorescence. Images are false-colored for fluorescence intensity. Images were captured at 2 images per second for 5 seconds prior to, and 35 seconds after photoactivation, and displayed at 2 frames per second. Movie frames reproduced in Fig. 4C.

**Movie S2. Localization of Rac1 activation is perturbed in caveolin or Coro1C knockdown fibroblasts.**  Rac1 activity distribution was detected using a Raichu-Rac FRET probe in cells spread on 50K before addition of H/0 (white flash). Movie captured at 1 frame every 2 minutes, for 11 minutes prior to, and up to 49 minutes after stimulation. Images are false-colored for FRET intensity. Movie frames reproduced in Fig. 6G.

**Movie S3. Caveolin expression is necessary for processive migration.** Control, caveolin-knockdown and *Sdc4* -/- MEFs migrating through a cell-derived matrix. Movie captured with a 5x lens at 1 image every 10 minutes for 10 hours.

**Movie S4. Shunting migration of caveolin-knockdown MEFs is not due to a tail retraction defect.** b_1_-integrin-GFP-expressing MEFs transfected with control or RCC2-targeted antisense oligo were filmed migrating through a cell-derived matrix to allow the rearmost attachment point to be seen. Movie captured at 1 frame every 10 minutes for 10 hours.
